# The association between early marriage and mental disorder among young migrant and non-migrant women: a Norwegian register-based study

**DOI:** 10.1186/s12905-022-01836-5

**Published:** 2022-06-27

**Authors:** Kamila Angelika Hynek, Dawit Shawel Abebe, Aart C. Liefbroer, Lars Johan Hauge, Melanie Lindsay Straiton

**Affiliations:** 1 Division for Mental and Physical Health, Norwegian Institute of Public Health, PO Box 222, 0213 Skøyen, Oslo Norway; 2 Faculty of Health Sciences, Oslo Metropolitan University, Oslo, Norway; 3 Department of Nursing and Health Promotion, Oslo Metropolitan University, Oslo, Norway; 4 Netherlands Interdisciplinary Demographic Institute, PO Box 11650, 2502 AR The Hague, The Netherlands; 5 Department of Epidemiology, University Medical Centre Groningen, University of Groningen, PO Box 30001, 9700 RB Groningen, The Netherlands; 6 Department of Sociology, Vrije Universiteit Amsterdam, De Boelelaan 1105, 1081 HV Amsterdam, The Netherlands

**Keywords:** Early marriage, Mental disorder, Migrant women, Outpatient mental healthcare service use, Registry data

## Abstract

**Background:**

Marriage is considered beneficial for mental health when stable and of high quality. Yet, it is unclear whether marriage is equally advantageous for everyone regardless of marital timing or migrant background. This study aimed to investigate the association between early marriage and mental disorder, defined by outpatient mental healthcare (OPMH) service use, and whether the association varies between migrant and non-migrant women.

**Methods:**

Using data from four Norwegian national registers, we applied discrete-time logistic regression analyses to study the aims of interest, among 602 473 young women aged 17–35 years. All women were followed from 2006 or the year they turned 17, and until first OPMH consultation, 2015 (study end), the year they turned 35, when emigrated, died, or changed marital status from married to separated, divorced, or widowed.

**Results:**

Results show that unmarried and early married women had increased odds of mental disorder when compared to on-time married women. However, the differences between the early and on-time married women were explained by differences in educational level. There was no significant interaction between marital status and migrant background.

**Conclusions:**

Differences in mental health between early- and on time married women are attributed to poorer educational attainment of women who marry early. Furthermore, migrant background seems to have a limited role in the association between marital timing and mental disorder. The promotion of formal education among young women could contribute to the accumulation of socioeconomic and psychosocial resources, thus, reducing the risk of mental disorder, also among early married women.

**Supplementary Information:**

The online version contains supplementary material available at 10.1186/s12905-022-01836-5.

## Background

Entering a marital union is, for many, one of the most important decisions in life and a step into adulthood. Age upon first marriage differs largely across countries. However, during the past decades the average age at first marriage has increased all around the world, among men and women alike [1]. Similarly, the age at which young people start to establish their position in the labour market, finish their education, move out of the parental home, and enter marital union, has moved from the early 20s to the late 20s and early 30s [2, 3]. Postponement of marriage to a later age can be advantageous, especially for women, due to an extended time for gaining more education and a stable income prior to marriage [4]. In many Western countries, marriage during the early 20s has therefore become unusual and can be seen as something unsound or non-normative [5, 6].

Older age at first marriage may also be a result of the increasing popularity of cohabitation, rather than marriage, as a preferred form of first union in several Western countries [2]. In Norway for instance, large differences between the non-migrant and migrant population in the preferred form of first union have, however, been documented. According to Wiik [7], cohabitation as first union was chosen by 94% of individuals without migrant background, while the numbers were 64% and 75% for descendants of migrants and migrants who migrated prior to the age of 18, respectively. Thus, marriage as a first union has become unusual in Norway and age at first marriage has been postponed from an average age of 25.8 years for women in 1989 to 33.1 years in 2019 [8].

Marriage at a later age is associated with greater relationship stability due to maturity of the spouses, and may be beneficial for mental health [9]. Early marriages, on the other hand, are less stable and at greater risk of divorce [10]. Thus, early marriage may not be beneficial or could even be detrimental to mental health, certainly compared to marriage at later ages [11]. However, it is unclear whether early marriage is associated with poorer mental health among migrant women in a European context. As migrant women are at increased risk of mental disorder [12] and some migrant groups tend to marry earlier than the general population [13, 14], it is of interest to examine whether the association between early marriage and risk of mental disorder differs between migrant and non-migrant women.

### Marital union and mental health

On average, entering a marriage can have a protective effect on one’s mental health, compared to remaining unmarried, however, it is not universally beneficial for all [15]. Stable and established marriages are found advantageous for mental health and well-being, relative to other, less committed relationship forms such as cohabitation [9, 16]. The positive effect of marriage on mental health is attributed to the social, emotional and financial support gained when entering a union, suggesting a causation effect [17]. Thus, married individuals are better able to cope with stress and other psychological challenges [18]. Yet, some evidence points in the direction of a selection effect; those who are happier and healthier are more likely to marry [16, 19]. Those with, or who are vulnerable to developing, mental disorders are at increased risk of either entering marriage at an early age or not entering marriage at all, and they have lower probability of marrying on-time or late [20–22]. However, stronger associations are found for causation rather than selection [16].

Whether marriage is beneficial for one’s mental health or not may depend on the timing of marriage. There is evidence suggesting that women who marry early (defined as before the age of 18 [23] and before the age of 26 [24]) report higher levels of depressive symptoms and more partner violence than those who marry later [23, 24]. Further, individuals marrying at earlier ages report more distress as compared to those marrying at later ages, but differences between the groups are mainly explained by the selection of more distressed individuals into early marriage [6]. There are, however, other studies suggesting that being in any kind of romantic relationship, both non-marital and marital is beneficial for mental health, regardless of age of entering such unions [6, 25], when compared to remaining single. Furthermore, Uecker [6] found no differences in life satisfaction between those entering marriage in their mid-20s compared to those entering marriage in their early 20s or as teenagers. Nevertheless, these studies come from non-European countries, and to the best of our knowledge, there is lack of European studies that investigated the association between marital timing and mental health.

However, it is not necessarily early marriage itself that is a risk factor for mental health, but also other aspects associated with early marriage. Economic hardship, lower educational attainment of spouses or less social support may increase the risk of mental disorders [26]. For instance, early marriages are more common among those from lower socioeconomic backgrounds and among those with low educational attainment [27, 28]. The timing of union formation and the preferred form of it can also depend on parental education. Individuals with highly educated parents are more likely to enter marriage or other union forms later than those with lower-educated parents [29]. Parental influence on partner choice and union type in some migrant groups may be present but it is greatest in families with poorly educated parents [30]. Increasing educational attainment of the child also results in more independent choices and later age at first marriage. The low socioeconomic status of the early married can play a role in the development of mental disorders, as low education, poor workforce participation–mainly unemployment–, and low household income are risk factors for mental disorder [31]. It is therefore plausible to assume that early marriage is less beneficial than on-time marriage because those entering early marriages are more likely to have lower levels of education, and that it could even be less beneficial than not being married during young adulthood.

To what extent the beneficial effect of marriage on mental health is generalizable to all groups in a society has been questioned by Roxburgh [32]. Findings in her study showed that for affluent Black American women, marriage has a detrimental effect on mental health (depression), while for White American women, marriage has a positive effect on mental health when compared to their non-married counterparts [32]. This study shows that differences in the relationship between marriage and mental health exist across sub-groups of women within a society. Thus, although the ethnic composition of the European population differs, it is possible that there could be differences in the association between early marriage and mental disorder by different migrant sub-groups in Europe. There are several possible reasons why the association could differ by migrant background.

One argument can be found in the life course perspective. The life course perspective suggests that psychological or social exposures during the different phases of life may have a long term impact on the risk of disease and other life outcomes in the future [33]. Furthermore, it is not only an event itself that is associated with positive or negative consequences but also its timing. Non-normative transitions has been found to negatively affect health [34]. Its timing may therefore be crucial for whether marriage will have positive or negative consequences for one’s mental health and well-being. However, what is considered as normative in some migrant groups, may be non-normative in the general population. A study conducted by Wiik [35] showed that individuals born in Norway to migrant parents (descendants) marry earlier than their non-migrant counterparts. Furthermore, there are large differences within the migrant group regarding the type of union and its timing [7]. Several European studies found that migrants, and migrant women in particular from South Asia, Eastern Europe and Middle East and North Africa, including Turkey marry directly and at earlier ages than the majority population [e.g., 7, 13, 14, 36]. Migrants who marry at a younger age have more ethnically homogamous unions, while individuals who marry later in life are more likely to have a partner with a different ethnic background than themselves [7, 35, 37, 38]. Wiik [7] suggested that more religious migrants, originating from Non-Western countries are more likely to follow the marital pathways and traditions from the country of origin, even if they were born in, or migrated to Norway as children or teens. This may result in less stigma and more acceptance of early marriage among some migrant groups, reducing the risk of poor mental health among those who marry early. For groups where marriage is postponed into the early 30s, such as for many Western migrants and Norwegians, there may be less acceptance of early marriages, since they deviate from the norm. This could increase the risk of poor mental health among those who marry early.

An alternative argument for why the association may differ by migrant background may relate to differing expectations about married women’s roles. Many migrant women originate from countries where a patriarchal structure dominates with men being the breadwinners and women being the homemakers. This may result in, for instance, poorer labour market participation of migrant women [39] particularly from African- and Asian countries [40], and increased risk of social isolation. Social isolation as a result of marriage, and perhaps early marriage in particular, may thus result in poor mental health among migrant women [41].

### Current study

Whilst marriage can have a protective effect on one’s mental health [42], early, non-normative marriage may be associated with poorer health among women [11]. Based on the presented evidence from the literature, we aim to investigate whether early marriage is associated with increased risk of mental disorder, defined by use of outpatient mental healthcare (OPMH) services, when compared to women who marry on-time and those who remain unmarried. We hypothesise that:

#### *Hypothesis 1*

Early marriage may be less protective of mental disorder than on-time marriage, but still more protective than remaining unmarried.

Furthermore, as previous research pointed at the increased possibility of early marriage and the increased risk of mental disorders among lower educated individuals, we hypothesize that:

#### *Hypothesis 2*

The potential differences between the early married and the on-time married will be partially explained by educational level.

There is also evidence suggesting that migrant women differ from majority women both in terms of marital timing, marital patterns and the risk of mental disorder development. Therefore, the second aim of this study is to investigate whether there are differences between migrant and non-migrant women in the association between marital status and mental disorder. We hypothesise that:

#### *Hypothesis 3*

There will be a difference in the strength of the association between marital timing and mental disorder across different migrant groups compared with majority women.

It can be *(a)* weaker among groups where early marriage is more normative but on the other hand *(b)* stronger among groups with traditional gender roles and increased risk of social isolation. Differences in the association may be largest for women from Asian- and African countries compared with majority women.

## Methods

### Data sources

This study is a dynamic, population-based cohort study utilizing data from four Norwegian registers. This means that individuals included in the study population could enter or leave the study at different time points. The registers were combined by use of a de-identifiable version of a unique personal identification number, assigned to all Norwegian citizens at birth, and to individuals registered as residents in Norway for at least six consecutive months. Demographic information such as year of birth, country of origin, migrant background, marital status, and year of marriage was extracted from the Central Population Registry. The National Database for the Reimbursement of Health Expenses (KUHR) was used to identify contact with OPMH services, used as a proxy for mental disorder. This database was available for years 2006–2015 and includes information on compensation claims from health professionals, including those working in OPMH services. The women’s highest education level was obtained from the National Education Database. Using a unique family number, we were also able to identify parents of the individuals and obtain information on parental education. Information regarding reception of child benefits was extracted from FD-trygd database. All measures are recorded annually.

### Study population

This study focuses on women aged 17–35, born between 1972 and 1997, who resided in Norway for at least two consecutive years between 2006 and 2015. The follow-up period started in 2006, or the year one turned 17. We followed each woman until the outcome of interest occurred—OPMH consultation. Otherwise, they were censored in 2015 (study end), the year they turned 35, emigrated or died or when they changed their marital status from married to divorced, separated, or widowed. We included only women born in Norway (majority and descendants) and those who migrated to Norway prior to the age of 18. Furthermore, we restricted our study population to women who got married in 2006 or later to have control over any potential OPMH consultations prior to, or around the time of marriage, as the data on OPMH service use are only available from 2006. The study population consists of 602 473 women.

## Measures

### Outcome

Our dependent variable, a proxy for mental disorder, is at least one OPMH consultation during the period 2007 and 2015. Individuals with OPMH consultation in 2006 or the first year they met requirements for study inclusion were excluded to increase the possibility that OPMH contact during the follow-up period was the first.

The Norwegian healthcare system is publicly funded, with all residents who are covered by the health insurance system having access and right to it. However, it is not free, with a small user fee that needs to be paid by individuals aged 18 or older. All user fees that exceed 2460 Norwegian kroner (NOK), which approximates ten GP consultations, are covered by the insurance scheme [43]. OPMH services are available throughout the country [44]. To access them, a referral from a general practitioner (GP) or psychologist is required. While mild to moderate mental disorders are usually treated by a GP at primary healthcare level [45], more severe mental disorders are treated at the specialist level. OPMH services constitute the majority of contacts at the specialist level in Norway. Inpatient care constitutes only around five percent of specialist mental healthcare use [46]. In this study, only information about OPMH contacts were available.

### Exposure

Marital status has three categories: unmarried, early marriage and on-time marriage. We applied a different cut-off for early marriage for the different migrant groups, based on the group’s average age at first marriage. This strategy was applied due to the differences in age at first marriage between the investigated groups. Thus, use of the same cut-off value would be inappropriate. Early marriage was defined as two years or more below the mean age at first marriage for the sub-group of interest. All marriages later than this cut off were defined as on-time and used as the reference category. The two-year limit was found to be the most optimal to achieve an adequate number of observations per category for each group to run a meaningful analysis. The same boundary was used by Zoutewelle-Terovan and Liefbroer [47] in order to define off-time events in terms of family formation and cohabitation. Marital status is a time-varying variable and only marriages entered at the age of 18 or later, and between 2006 and 2014 were included in the study. Mean age at first marriage with standard deviation (SD) for each group are shown in Table 1.

**Table 1:** Characteristics of the study population by migrant background and region of origin

	Total	Migrant background		Region of origin							
		Majority^a^	Migrant	Nordics	Western Europe	EU Eastern Europe	Non-EU Eastern Europe	MENA	Sub-Saharan Africa	South Asia	E/SE Asia
N of observations	4 126 573	3 859 861	266 712	15 793	12 196	19 358	39 113	49 053	36 381	53 197	41 621
N (%) of individuals	602 473	560 576 (93.0)	41 897 (7.0)	2480 (0.4)	2243 (0.4)	3512 (0.6)	5676 (0.9)	7673 (1.3)	6086 (1.0)	7836 (1.3)	6391 (1.1)
Years in study, mean (SD)	6.8 (2.9)	6.9 (2.9)	6.4 (2.9)	6.4 (3.0)^b^	5.4 (2.9)^b^	5.5 (2.8)^b^	6.9 (2.9) ^ns^	6.4 (2.9)^b^	6.0 (2.9)^b^	6.8 (2.9)^ns^	6.5 (2.9)^b^
Age, mean (SD)	27.4 (5.9)	27.6 (5.9)	24.3 (5.1)	25.8 (5.9)^b^	23.4 (5.4)^b^	23.1 (5.2)^b^	24.8 (4.9)^b^	23.9 (4.7)^b^	23.4 (4.8)^b^	24.6 (4.9)^b^	25.1 (5.6)^b^
Mean age at marriage (SD)	28.5 (3.8)	28.7 (3.7)	25.3 (3.9)	28.7 (3.8) ^ns^	27.0 (4.5)^b^	26.5 (4.3)^b^	24.6 (3.7)^b^	23.9 (3.7)^b^	24.7 (3.8)^b^	25.1 (3.4)^b^	27.6 (3.5)^b^
Marital status				^b^	^b^	^b^	^b^	^b^	^b^	^b^	^b^
Unmarried	84.1	84.1	85.8	88.1	92.9	91.5	79.9	80.3	92.2	75.3	86.1
Early	4.7	4.7	4.6	3.6	2.3	3.0	7.1	6.3	2.6	8.8	4.1
On-time	11.2	11.3	9.6	8.4	4.8	5.5	13.0	13.4	5.2	15.9	9.0
OPMH use				^ns^	^b^	^b^	^c^	^ns^	^b^	^b^	^b^
Yes	12.3	12.5	9.8	12.5	9.3	8.1	10.9	13.3	9.0	8.4	7.1
Education				^b^	^b^	^b^	^b^	^b^	^b^	^b^	^b^
≤ Compulsory	25.8	24.6	41.5	29.5	32.5	48.1	37.0	45.9	53.9	35.8	39.8
Upper-secondary	32.7	33.0	27.7	27.1	25.6	23.3	30.6	28.7	22.6	20.9	28.5
Tertiary	40.7	42.0	23.7	30.5	22.9	19.8	30.0	20.2	13.0	28.9	26.2
Unknown	0.9	0.4	7.0	12.9	19.0	8.8	2.4	5.2	10.5	4.5	5.5
Motherhood				^b^	^b^	^b^	^b^	^b^	^b^	^b^	^b^
Yes	35.9	37.1	19.9	26.4	12.5	12.8	22.9	19.1	24.2	17.5	20.9
Parental education				^b^	^b^	^b^	^b^	^b^	^b^	^b^	^b^
≤ Compulsory	59.7	9.1	16.6	3.0	3.4	2.3	9.5	26.1	10.6	27.2	21.7
Upper-secondary	46.6	48.7	19.0	13.7	10.4	18.5	28.2	18.2	12.7	23.5	18.0
Tertiary	38.9	40.1	22.3	32.7	34.5	22.1	28.0	23.2	14.2	22.8	15.0
Unknown	4.9	2.1	42.1	50.7	51.7	57.2	34.3	32.5	62.5	26.5	45.3
Cohort				^b^	^b^	^b^	^b^	^b^	^b^	^b^	^b^
1972–1977	15.3	16.2	3.2	8.8	5.8	3.6	1.9	1.5	2.0	1.7	6.1
1978–1982	17.1	17.8	8.3	14.8	6.9	7.0	8.8	6.4	5.8	8.4	11.3
1983–1987	20.2	20.5	18.4	20.5	13.2	11.9	21.6	19.8	16.1	21.2	16.8
1988–1992	23.6	23.2	29.3	27.6	26.0	20.6	34.1	32.3	27.0	31.4	27.6
1993–1997	23.8	22.5	40.9	28.3	48.1	56.9	33.6	40.1	49.2	37.2	38.2

*E/SE* Asia–East/South East Asia; *EU* European Union; *MENA* Middle East and North Africa

^*^All measures are for the last year individuals are in the study

^ns^Non-significant

^a^Reference group

^b^ < .001

^c^ < .01

### Sociodemographic covariates

We divided our sample into migrant (born in Norway or abroad with two foreign-born parents) and majority women (born in Norway or abroad with at least one Norwegian born parent). Migrant women were further divided into eight groups according to their region of origin: Nordics, Western Europe, European Union (EU) Eastern Europe, non-EU Eastern Europe, Middle East and North Africa, including Turkey (MENA), Sub-Saharan Africa, South Asia and East/ South East (E/SE) Asia. Women originating from the Americas and Oceania were excluded from the study population, since there were too few in each region.

Further, own educational attainment, as well as parental educational attainment were used to indicate the socioeconomic status of the individual. Own educational attainment is a time-varying variable measured for each year the individual is in the study, while parental education is measured when the woman is aged 16 and is a time-invariant variable. Both variables have four categories: compulsory education or less (≤ compulsory), upper-secondary education, tertiary education, and unknown education. Motherhood (yes/no) was based on whether the woman was receiving child benefit or not. Child benefit is commonly entitled to mothers who have a caring responsibility for a child from two months after a child’s birth until a month before a child turns 18 years. It is an automatic payment, regardless of income level [48]. Age and age squared (age^2^) are both continuous and time-varying variables. Age^2^ is used to estimate the potentially non-linear association between OPMH service use and age. We also control for potential cohort-effect by categorizing birth cohorts into five years intervals: 1972–1977, 1978–1982, 1983–1987, 1988–1992 and 1993–1997. This variable is time-invariant. Accounting for a possible cohort-effect in the analysis is important as the normative age at first marriage might differ for the various cohorts due to the changing marital patterns and increasing age at first marriage [1].

## Statistical analysis

Chi-square test and one-way ANOVA were used to compare the characteristics of the migrant groups with majority women being used as a reference group. All characteristics are measured the last year individuals contributed to the study. By using discrete-time (logistic regression) analysis [49], we study the yearly odds of mental disorder, defined by outpatient mental healthcare services use, among women who married on-time (reference category), early or remained unmarried. We also consider differences between majority and migrant women. The use of discrete-time analysis is more appropriate than a continuous-time analysis as the events are measured at discrete-time points [50]. To investigate the differences by migrant background, we introduce an interaction term between marital status and migrant background.

The datafile was organized into person-period format, where each year an individual is at risk is represented by a separate record. Our data are right censored with a non-informative censoring. Thus, we assume that individuals in our dataset who are excluded throughout the study period are not at greater or lesser risk of experiencing OPMH consultation (failure) than those remaining in the study until the end of follow-up period in 2015. The results from a discrete-time (logistic regression) analysis are presented in four models. Model 1 is a base model including only the main exposure, marital status, and with controls for age, age^2^ and cohort. In model 2, we control additionally for own education. In model 3, we control for migrant background, parental education, and motherhood. In the final model (model 4), we include the interaction term between marital status and migrant background. The results are presented as yearly odds ratios (OR) with 95% confidence intervals (95% CI). Due to a large percentage of missing values on parental education and some missing values on own education, especially among migrants, we conducted a sensitivity analysis with complete cases only (results available on request). The results were comparable to those presented in Table 2, with no visual changes in the main outcome. Thus, the main analysis is run with inclusion of the unknown category to ensure the size of the groups and the statistical power of the analysis. All the analyses were conducted in Stata 17.0.

**Table 2:** Discrete time analysis for the association between early marriage and outpatient mental healthcare service use. Odds ratio and 95% confidence intervals

	Model 1				Model 2				Model 3				Model 4			
	OR	95% CI		p-value	OR	95% CI		p-value	OR	95% CI		p-value	OR	95% CI		p-value
*Marital status*																
On-time	1.00				1.00				1.00				1.00			
Unmarried	1.28	1.23	1.33	^a^	1.15	1.11	1.20	^a^	1.13	1.09	1.18	^a^	1.14	1.10	1.19	^a^
Early	1.15	1.10	1.22	^a^	1.00	0.95	1.05	^ns^	0.99	0.94	1.04	^ns^	0.99	0.94	1.05	^ns^
*Migrant background*																
Majority									1.00				1.00			
Non-EU Eastern Europe									0.67	0.61	0.73	^a^	0.73	0.56	0.97	^c^
MENA									0.80	0.75	0.86	^a^	0.90	0.73	1.11	^ns^
South Asia									0.52	0.48	0.56	^a^	0.56	0.44	0.71	^a^
Interaction marital status* *migrant background*																
Unmarried* Non-EU Eastern Europe													0.89	0.66	1.19	^ns^
Unmarried* MENA													0.88	0.71	1.10	^ns^
Unmarried* South Asia													0.91	0.70	1.18	^ns^
Early* Non-EU Eastern Europe													1.15	0.76	1.73	^ns^
Early* MENA													0.82	0.58	1.16	^ns^
Early* South Asia													1.03	0.72	1.49	^ns^

Number of person-years 4 001 224; Number of individuals 581 109

Model 1 adjusted for age, age^2^ and cohort; Model 2 adjusted for model 1 and education; Model 3 adjusted for model 2 and migrant background, parental education, and motherhood; Model 4 adjusted for model 3 and interaction between marital status and migrant background

*E/SE* Asia East/South East Asia; *EU* European Union; *MENA* Middle East and North Africa; *OR* odds ratio; *95% CI* 95% confidence interval

^ns^Non-significant

^a^p < .001

^b^p < .01

^c^p < .05

## Results

### Descriptive statistics

The characteristics of the study population are presented in Table 1, for the majority women and migrant women, but also separately for migrant women by region of origin. The study population consisted of 93.0% of majority women and 7.0% of migrant women. The highest percentage of migrant women originated from MENA and South Asia.

All migrant groups had significantly lower age at first marriage than majority women, except from migrants from Nordics with mean age at first marriage of 28.7 years. Women with background from MENA, Sub-Saharan Africa and non-EU Eastern Europe had the lowest mean age at first marriage, 23.9, 24.6 and 24.7 years respectively. Regarding marital status, most women were still unmarried at the end of the study period. The highest proportion of early married women was among women originating from South Asia (8.8%) followed by women from non-EU Eastern Europe (7.1%) and MENA (6.3%). The highest use of OPMH services was among women from MENA (13.3%), Nordics (12.5%) and majority women (12.5%). Both of these migrant groups did not significantly differ from majority women regarding OPMH service use. OPMH service use was significantly lower for all other migrant groups as compared to majority women, with the lowest use among women from EU Eastern Europe (8.1%) and E/SE Asia (7.1%). The descriptive statistics for the remaining covariates by migrant background and region of origin are presented in Table 1.

When combining the information regarding OPMH service use and marital status, the number of observations for some groups were relatively small (see Additional file 1). Therefore, to ensure the statistical power of the analysis presented in Table 2, we conducted the discrete-time analysis focusing only on the groups with the largest number of observations per group when combining information on marital status and OPMH service use–Majority, non-EU Eastern Europe, MENA and South Asia. The selection of these four groups derived an analytical sample of 581 109 women and 4 001 224 person-years. The number of individuals using OPMH services were 72 490 for the four groups.

### Discrete-time analysis

Table 2 shows the results of the discrete-time analysis, applied to investigate whether early marriage is less protective of mental disorder, defined by use of OPMH services, than on-time marriage, but still more protective than remaining unmarried (*Hypothesis **1*). The results from the base model 1 showed that unmarried women and those who marry early have higher odds of OPMH service use, when compared to women who marry on-time, OR = 1.28 (95% CI 1.23–1.33) and OR = 1.15 (95% CI 1.10–1.22) respectively. In model 2, we controlled for own education, to investigate, whether the potential differences between the early married and the on-time married could be explained by educational level (*Hypothesis **2*). Results in model 2 suggest that remaining unmarried was associated with increased odds of OPMH service use, though the association was somewhat weaker, while the differences between early and on-time married women were non-significant. Following additional adjustment for migrant background, parental education and motherhood (model 3), the association between marital status and OPMH service use remained virtually unchanged. In fully adjusted model 4, we introduced an interaction term between migrant background and marital status in the model (*Hypothesis **3*). Results from interaction analysis showed no significant difference by migrant background in the association between marital status and OPMH service use.

## Discussion

By using a life course perspective, this study aimed to investigate the association between early marriage and mental disorder, defined as OPMH service use, with a focus on the differences between migrant and non-migrant women. As suggested by the life course perspective, exposures during the various phases of life may impact health in positive or negative ways by increasing the risk of disease and other life outcomes [33]. Non-normative events, such as early marriage, can have a negative impact, therefore, it is not only an event itself that may influence health outcomes, but also its timing [34]. We therefore hypothesized that early marriage may be less protective for mental disorder than on-time marriage, but still more protective than remaining unmarried (*Hypothesis **1*). This hypothesis was supported by the analysis. We found that women who married early had significantly higher odds of mental disorder when compared to on-time married women. This was also true for unmarried women who had elevated odds of OPMH service use when compared to on-time married women, but also higher odds than early married women. However, after accounting for educational level, this difference between early and on-time married women was no longer significant. This supports *Hypothesis **2*, that the potential differences between early married and on-time married women could be explained by educational level. Previous research found that postponement of marriage to later ages is common among higher educated women [11], while those marrying early have lower educational aspirations [3]. Education level is also associated with risk of mental disorders, with those with the lowest education being at the highest risk [31]. Thus, the postponement of marriage to a later age may be beneficial with regards to gaining more education and thus lowering the risk of mental disorders later in life. However, it is important to stress that there may be many other reasons for why postponement of marriage may be beneficial for one’s mental health that we were unable to account for. For instance, marriages entered at later ages has been found to be more stable as a result of the maturity of individuals entering such unions [9]. Additionally, those entering early marriages often have lower socioeconomic status than those who marry at later ages, which may further play a role in the development of mental disorders. Previous research found that in addition to low education, poor workforce participation and low household income constitute risk factors for mental disorder development [31].

Another important finding of this study is the increased risk of mental disorder among women who remain unmarried. This finding is in accordance with our hypothesis of the protective effect of marriage on mental health (*Hypothesis **1*), and with previous research from the U.S., showing that being in a marital relationship, regardless of the timing and form of it, is beneficial for mental health compared to single individuals [e.g., 6, 25]. However, previous research also indicates that individuals with poor mental health may have lower chances of marriage or increased probability of early marriage [18, 22]. Thus, we cannot rule out selection of individuals with poor mental health into marriage in general or early marriages in particular. In other words, good mental health could be a prerequisite for marriage, rather than a result of it.

Further, we examined differences in the link between the timing of marriage and mental health between migrant and majority women. Previous research pointed to the ethnic homogamy of early marriages among individuals with migrant background [e.g. 35, 37]. Thus, on one hand, we hypothesized that migrant women who marry early could benefit more than their non-migrant counterparts, as they may be met with less stigma and more acceptance when entering a marital union, regardless of the timing, than early marrying majority women (*Hypothesis 3a*). On the other hand, for women originating from countries with a more traditional gender role distribution, early marriage may result in an increased risk of social isolation and thus, also increased risk of mental disorder (*Hypothesis 3b*). The poorer labour market participation of women mainly originating from non-Western countries following the marriage [39], coupled with the poorer educational attainment of women who marry early [27] could lead to greater social isolation and increased risk of mental disorders among women with migrant background [41]. However, we found no difference in the association between timing of marriage on OPMH service use between majority women and the three migrant groups we investigated, thus not rendering support to either hypothesis. Although the studied groups may differ in what constitutes on-time and early marriage, the link between the timing and mental health is the same for the studied migrant groups and for non-migrant women. Furthermore, early marriage itself is not associated with disorder, but rather that those who marry early often have lower educational attainment, making this group of women more vulnerable to mental disorders.

### Limitations

The findings of this study should be interpreted with consideration of the following limitations. The main limitation of this study is the focus on marital union only and no other union forms such as cohabitation. The increasing popularity of cohabitation as a preferred form of first union in many Western countries [2], may result in fewer formal marital unions among the majority population. In this study, we focussed on marital union only as we did not have data on informal unions such as cohabitation or non-cohabiting relationships. Thus, our unmarried category includes both single individuals and those in cohabiting and non-cohabiting unions, a heterogeneous group. Furthermore, there is research suggesting that, cohabitation enhances well-being no less than marriage [51, 52], while other studies suggest marriage is more beneficial for mental health than cohabitation [9, 16]. By treating cohabitants and singles as one group, we may be underestimating the mental health differences between unmarried and on-time married individuals. This may particularly be the case for non-migrants where cohabitation is often the preferred choice of first union [7]. Despite the increase in cohabitation also among migrants and descendants in the recent years [7], there is likely to be a difference across our four groups in the proportion of unmarried individuals who are in cohabitating relationships.

Despite the objectivity and national coverage of the registers applied to identify women using a relatively high threshold outcome as a proxy for mental disorder—namely OPMH services—we only detect individuals who sought help and not all women experiencing mental disorders. Additionally, our outcome variable, OPMH service use, does not cover other contacts at specialist level such as inpatient care. However, as indicated earlier, inpatient care constitutes only a minor share of specialist mental healthcare use [46]. Further, the onset of a mental disorder is likely to occur sometime prior to OPMH contact, meaning that some women may have had a mental disorder, or mild to moderate mental health problems that could have been treated at primary care level in the years preceding the study or prior to entering a marriage. This limits our ability to draw causal conclusions about the direction of the relationship between marriage and lower risk of mental disorder.

Furthermore, as we were unable to include all migrant groups in the analysis, the results based on the included groups are not generalizable to migrants overall, but rather to the women originating from the investigated regions. The generally lower percentage of migrant women using OPMH services as compared to majority women, except from women from Nordics and MENA, provides a justification to speculate that migrant women may face barriers in the health system that prevent them from seeking help. Previous studies suggested that stigma related to mental health, low mental health literacy and unfamiliarity with the healthcare system may hinder migrants from mental healthcare seeking [53, 54]. However, use of registers may still be a better way of studying migrant health and risk factors associated with it when compared to surveys that often suffer from low response rates [55].

Additionally, to ensure the statistical power of the analysis, we did not differentiate between migrants and descendants in this study. However, we chose to only include migrants who migrated as teens or children, thus assuming that those who migrate and those born to migrant parents would be quite similar regarding their marital preferences, including the timing of the marriage [35]. Lastly, in this study, only few control variables could be included in the analysis. Potential additional factors that could help explain the relationship between marital timing and mental disorder include for instance, desired age at marriage, social support, income, or labour market attachment. Due to the limited types of variables available in registries, the former two factors were not available. Although income and labour market attachment are available in registries, these may be a poor measure of socioeconomic status for the studied population, as many young women are still in education or are establishing their position in the labour market.

## Conclusions

This study shows that marriage is beneficial for women’s mental health regardless of its timing, when compared to those who remain unmarried, once educational attainment is taken into account. To reduce the risk of mental disorder among individuals who marry early, it is important to promote education among young women. For many, entering a marital union at a young age may result in less time to gather socioeconomic resources. Education increases the possibilities in life through the accumulation of social capital, contributes to the development of important skills necessary to cope with daily hassles and help seeking at early stages of mental disorder. Our results show that the differences in educational level explain the higher odds of mental disorders among those who marry early when compared to those who marry on-time. Furthermore, our results show no differences in the association between marital timing and mental disorder between migrant and majority women, indicating the limited role of migrant background in this association. Further research should aim to investigate other groups of migrants and if possible, also investigate the effect of early and on-time cohabitation for migrants and non-migrants.

## Supplementary Information

**Additional file 1**.** Table 1**. Outpatient mental healthcare (OPMH) use by marital status and region of origin, number, and percentage of individuals per group.

## Abbreviations

95% CI: 95% Confidence interval
E/SE Asia: East and South East Asia
EU: European Union
GP: General practitioner
KUHR: The National Database for the Reimbursement of Health Expenses
MENA: Middle East and North Africa
NOK: Norwegian Kroner
OPMH: Outpatient mental healthcare
OR: Odds ratio
SD: Standard deviation
US: United States

## Declarations

### Availability of data and materials

The data that support the findings of this study are available from Statistics Norway and HELFO but restrictions apply to the availability of these data, which were used under license for the current study, and so are not publicly available. Data are however available from Statistics Norway and HELFO if ethical approval is granted. Requests to access these datasets should be directed to Statistics Norway (https://www.ssb.no/en/data-tilforskning/utlan-av-data-til-forskere) and HELFO (https://www.helsedirektoratet.no/tema/statistikk-registre-og-rapporter/helsedata-og-helseregistre/kuhr#sokomdatafrakuhr).
