# Supplementary material for: The association between early marriage and mental disorder among young migrant and non-migrant women: a Norwegian register-based study
Source: BMC Womens Health. 2022 Jun 27;22:258. doi: 10.1186/s12905-022-01836-5 (PMC9237981; doi:10.1186/s12905-022-01836-5)
Supplement: Supplementary file 1 — Additional file 1. Table 1. Outpatient mental healthcare (OPMH) use by marital status and region of origin, number, and percentage of individuals per group. [file 12905_2022_1836_MOESM1_ESM.docx]

**Additional file 1.**

Table S1 shows the number and percentage of individuals who did and did not use OPMH services, by marital status and by region of origin. The results show that OPMH service use by marital status varies between the studied groups. OPMH service use by marital status seemed to vary between the studied groups, though on-time married women had lower use in all groups except for those from Sub-Saharan Africa. For majority women, and migrant women from MENA and E/SE Asia, the unmarried group had the highest percentage with OPMH service use, followed by early married and on-time married women. The pattern was, however, different for the remaining migrant groups, where a higher percentage early married women had used OPMH service than their unmarried counterparts.

| Table S1 Outpatient mental healthcare (OPMH) use by marital status and region of origin, number, and percentage of individuals per group. | | | | | | | | | | | |
| --- | --- | --- | --- | --- | --- | --- | --- | --- | --- | --- | --- |
|  | **Total** | **Migrant background** | | **Region of origin** | | | | | | | |
|  |  | Majority ^2^ | Migrant | Nordics | Western  Europe | EU Eastern  Europe | Non-EU  Eastern  Europe | MENA | Sub-  Saharan  Africa | South Asia | E/SE  Asia |
| **OPMH by marital status** |  |  |  |  |  |  |  |  |  |  |  |
| Unmarried ^1^ |  |  |  | ^ns^ | ^a^ | ^a^ | ^a^ | ^ns^ | ^a^ | ^a^ | ^a^ |
| Yes | 67 784 (13.4) | 64 215 (13.6) | 3611 (10.0) | 284 (13.0) | 193 (9.3) | 261 (8.1) | 512 (11.3) | 853 (13.8) | 524 (9.3) | 526 (8.9) | 416 (7.5) |
| No | 438 699 (84.6) | 412 698 (86.4) | 32 413 (90.0) | 1900 (87.0) | 1891 (90.7) | 2952 (91.9) | 4024 (88.7) | 5310 (86.2) | 5085 (90.7) | 5375 (91.1) | 5141 (92.5) |
| Early ^1^ |  |  |  | ^ns^ | ^ns^ | ^ns^ | ^ns^ | ^ns^ | ^ns^ | ^ns^ | ^ns^ |
| Yes | 2764 (9.7) | 2540 (9.7) | 206 (10.7) | 13 (14.8) | 7 (13.5) | 10 (9.4) | 51 (12.7) | 60 (12.5) | 6 (3.8) | 62 (9.0) | 15 (5.7) |
| No | 25 625 (90.3) | 23 609 (90.3) | 1714 (89.3) | 75 (85.2) | 45 (86.5) | 97 (90.6) | 350 (87.3) | 422 (87.5) | 153 (96.2) | 627 (91.0) | 247 (94.3) |
| On-time ^1^ |  |  |  | ^ns^ | ^ns^ | ^ns^ | ^ns^ | ^a^ | ^ns^ | ^ns^ | ^ns^ |
| Yes | 3748 (5.5) | 3440 (5.4) | 286 (7.1) | 14 (6.7) | 9 (8.4) | 14 (7.3) | 56 (7.6) | 105 (10.2) | 18 (5.7) | 70 (5.6) | 22 (3.9) |
| No | 63 883 (94.5) | 56 737 (94.6) | 3752 (92.9) | 182 (93.3) | 93 (91.6) | 166 (92.7) | 634 (92.4) | 923 (89.8) | 300 (94.3) | 1176 (94.4) | 550 (96.1) |

^1^ N (%); ^2^ Reference group; ^ns^ non-significant; ^a^ p-value <.001; ^b^ p-value <.01; E/SE Asia – East/South East Asia; EU – European Union; MENA – Middle East and North Africa; OPMH – outpatient mental healthcare
